# Effects of Video Task With a High-Level Exercise Illustration on Knee Movements in Male Volleyball Spike Jump

**DOI:** 10.3389/fpsyg.2021.644188

**Published:** 2021-08-30

**Authors:** Xiangyu Liu, Huiming Huang, Xiaohan Li, Jianshe Li, Huijuan Shi, Aiwen Wang

**Affiliations:** ^1^Research Academy of Grand Health, Faculty of Sports Science, Ningbo University, Ningbo, China; ^2^Beijing Key Laboratory of Sports Injuries, Institute of Sports Medicine, Peking University Third Hospital, Beijing, China

**Keywords:** team sports, lower body, kinematic, kinetic, internal focus

## Abstract

Hazardous knee biomechanics, such as excessive knee affordance link with injuries in volleyball spike jumps (SPJs) and can be reconfigured by the enhancement of internal focus. The study aimed to explore the effects of video tasks illustrating a high-level SPJ on knee movement in the volleyball SPJ with 15 elite male volleyball athletes. This study investigated the knee movements in sagittal, coronal, and transverse planes before and after the video task in SPJ using one-dimensional statistical parametric mapping (SPM 1D) and discrete statistics. The SPM 1D indicated a larger knee flexion angle (31.17–73.19%, *t* = 2.611, and *p* = 0.012), increased knee flexion moment (19.72–21.38%, *t* = 0.029, and *p* = 0.029), and increased knee adduction angular velocity (49.07–62.64%, *t* = 3.148, and *p* = 0.004) after video task; alternatively, smaller knee external rotation angular velocity (45.85–49.96%, *t* = 5.199, and *p* = 0.017) and vertical ground reaction (vGRF) (3.13–5.94%, *t* = 4.096, and *p* = 0.014; 19.83–21.97%, *t* = 4.096, and *p* = 0.024) were found after the task. With discrete value statistics, the video task increased the peak of knee flexion angle while decreased the peak of extension moment, flexion moment, abduction moment, external moment, the first peak vGRF, and related loading rate.

Conclusions: The results indicate that knee biomechanics in volleyball SPJ positively influenced by the video task. The task has the athletes control the knee movements more actively and improves the original hazardous movement strategies. Therefore, the video task presumably can abate the occurrence of knee injuries in volleyball SPJ. Further validation especially in the exercise effect is needed in the future.

## Introduction

Volleyball is a popular Olympic sport wherein athletes have to perform complex actions. Spike jumps (SPJs) are the necessary skills in determining match results in volleyball (Costa et al., 2017). Unlike standing vertical jumps, the SPJ movement, such as run-up and braking before take-off (Coleman et al., 1993). Research has indicated that sports, such as cutting, stop-jump, landing, or sudden deceleration are commonly related to acute knee injuries (Coleman et al., 1993; Zahradnik et al., 2015; Yang et al., 2018; Mohd Azhar et al., 2019). The 90% of volleyball injuries occur in the lower extremity, especially in the knee as it is particularly vulnerable (Zahradnik et al., 2017), of which non-contact anterior cruciate ligament (ACL) tears (Kanamori et al., 2002; Noyes et al., 2015), osteoarthritis (Shen et al., 2013), and articular cartilage lesions (Dua et al., 2016) are the most prevalent. Studies have suggested that the hazardous biomechanics of lower limb in SPJ (a smaller knee flexion angle, increased extension knee moment, increased abduction knee moment, and higher vGRF) would result in more frequent knee injuries occurrence (Xu et al., 2021a). Even though many training protocols that aim to avoid knee injuries (stretching exercises, softer landing methods, more scientific warm-up activities, etc.) have been proposed, the rate of knee injuries in competitions is still relatively high (Yang et al., 2018). Therefore, further research on the training program for knee injury prevention is needed.

Recent studies have suggested that attention strategies could modify movement biomechanics (James and Wu, 2015). Furthermore, when directed to an internal focus, athletes changed their approaches to control the movements of their bodies more actively (Marchant et al., 2009; Peh et al., 2011). Video task as a potential way to improve internal attention has been observed to enhance the control of body movements (Oñate et al., 2005; Peh et al., 2011). Shimokochi et al., reported a 23% reduction of the peak of vGRF in female college volleyball athletes after 3 min of video task procedure when landing (Shimokochi et al., 2009). Parsons et al., reported the immediate improvement of kinematics in SPJ landing for female adolescent volleyball athletes after a video task procedure (Parsons and Alexander, 2012). However, the previous studies showed that male athletes performed differently compared to female athletes during an emergency stop and take-off (Etnoyer et al., 2013). There are gender differences in sustained attentional control and motor skills (Gromeier et al., 2017; Riley et al., 2017), and especially the effect of video tasks on the knee movements in volleyball SPJ in male athletes has not been reported yet.

Discrete value analysis was widely used in studies whereas the single value analysis as neglects the time characteristics through the landing or take-off phase cannot represent the curve (Krosshaug et al., 2007; Xu et al., 2021b). At present, the research on the time-space variability of biomechanics has made great achievements, and statistical parametric mapping (SPM) has been proposed (Pataky, 2010; Pataky et al., 2013; Xu et al., 2020). The SPM has been used in the field of biomechanics to determine the curve differences such as kinematics and dynamics throughout a movement cycle. Combining with the one-dimensional characteristics of joint movement over time in this research, the traditional discrete analysis is used to detect the biomechanical changes of knee movements in the volleyball spike, and more comprehensive validation whereby of the effect of video task on volleyball spike was concluded.

The current study aimed to compare the biomechanics of the knee in SPJ before and after the video task. We hypothesized that: 1) the vertical ground reaction (vGRF) impulse of volleyball spiking would increase after the video task; and 2) the knee load would be reduced after the video task, leading to a reduced risk of knee injuries.

## Materials and Methods

### Participants

In the study, 15 male volleyball athletes with injury-free (age 21.4 ± 0.83 years; height 188.8 ± 1.37 cm; body mass 79.4 ± 3.47 kg) were enrolled at a local university. They all played National College Volleyball League and won the top eight. The subjects recruited in this experiment were all national second-level volleyball athletes, and they had been training 5 times a week. To avoid different technical actions of offensive players in the different positions (outside hitter, opposite hitter, middle blocker, libero, and setter) confounding the results, all subjects were outside hitters. All the subjects were right-handed; they all were informed of all the experiment details and potential risks and signed an informed consent approved by the Institutional Review Board of the local University (Ningbo University, China). The eligible criteria for subjects were as follows: (1) no surgery situation/injury-free; and (2) no medical problems within 6 months before the test. All the subjects did not take similar tests and confirmed not to receive video task intervention before.

### Video Task

The video task adopted in the current study is an American professional spiking instructional video downloaded from the Internet. The focus of the video is upon lower limb exercises, such as run-ups, jumps (the right foot touches the ground first, and the left foot quickly catches up to act as a brake), kicks, and jumps. The video was composed of multiple combinations of the run-up and jumps spike and consisted of two parts, with the first part playing 1 min at normal speed and the counterpart playing 2 min at half normal speed (Parsons and Alexander, 2012). The video task was conducted by browsing the video once before the second test. The video task would be carried out after a break (10 min); they were instructed to a separate area in the laboratory without any disturbance around and watched a 3-min video alone and perform a volleyball spike experiment immediately after the task.

### Procedures

Basic information (height, weight, and age) was collected before the biomechanics assessment. The dominant limb was screened as the limb to kick a ball. The volleyball spiking line (e.g., large slash, small slash, and straight-line) was an important factor that affects the take-off of the smash. In addition, due to the limitation of laboratory space, in consideration of the more standard straight smash action and the easier scope of the smash route, therefore, the straight smash action at the fourth position was chosen.

**Figure 1:**
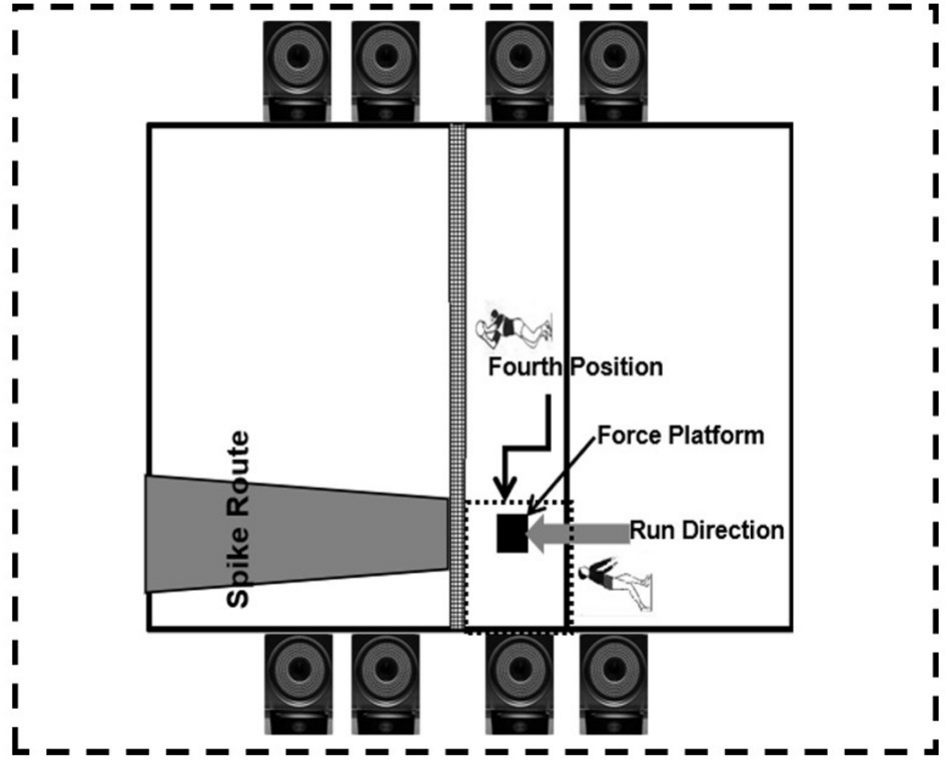
Illustration of experiment.

A mimicked volleyball court was set in the laboratory. The infrared high-speed system with eight cameras (Vicon, Oxford Metric Ltd., Oxford, UK, 200 Hz) was set around the court for kinematics collection; force platform (AMTI, Watertown, MA, United States, 1,000 Hz) was set in the center of the court was used for kinetics data collection; they were synchronized in the movement test (Figure 1). A referred marker set with 20 reflex markers (diameter, 12.5 mm) (Xu et al., 2021b) was adopted in the current study. A standing trial was collected for the moralized calculation at the beginning of the test.

**Figure 2:**
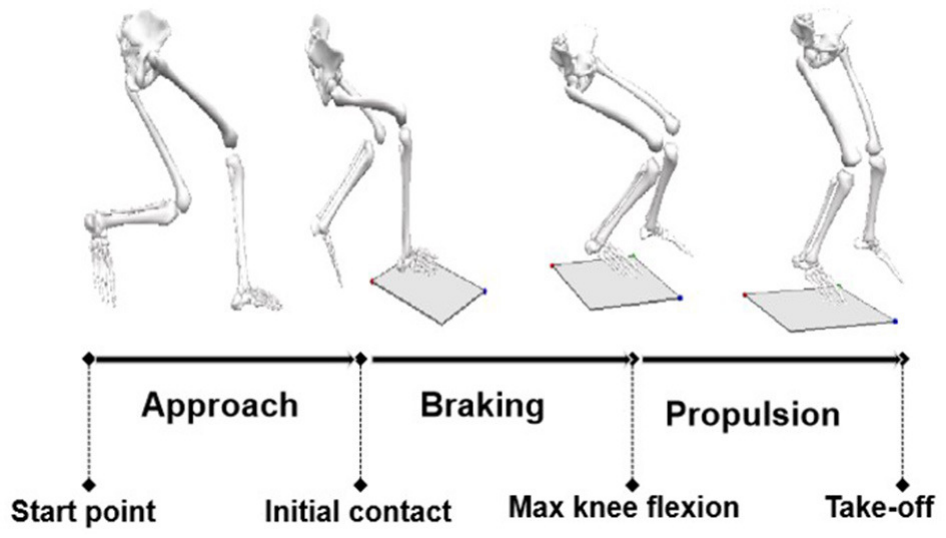
The process of jump following a spike.

After the warm-up procedure (8 min of light jogging and hitting a volleyball back and forth between partners), five familiar trials were performed in the testing area before the formal test. The subjects were asked to perform volleyball SPJ before and after the intervention, and a 30 min break was given for the purpose to avoid fatigue. The illustration of the SPJ is shown in Figure 2. There was a coach on-site to supervise the experiment in the test to ensure a qualified trial. The trials accepted had to meet the following criteria: (1) a natural and powerful trial judged by the coach; and (2) the ball landed in the target area (Figure 1). For each subject, five trials at least were collected before and after the video intervention.

### Data Processing

The original signals (marker coordinates and ground reaction force) were filtered by 7 and 15 Hz (lower pass, fourth-order Butterworth) respectively, calculated from the formulation reported by Bing Yu et al. (1999). A threshold of vGRF (10 N) was used to detect the initial point and tale-off point and the phase between them was defined as the SPJ. A hybrid rigid lower limbs model was used for the calculation of knee movement in commercial biomechanical software (Visual 3D, C-Motion, Bethesda, MD, USA). The jump height was calculated according to the local center of gravity of the subject within the software. The maximum jump height was measured before the biomechanics assessment, and the range with ±5% of the maximum jump height was used as the standard to evaluate the trials.

The interesting biomechanics variables on knee movement were calculated in the commercial software; SPM 1D calculation was performed in MATLAB with a custom MATLAB script. The Euler sequence (X–Y–Z) was adopted in the biomechanics processing (Cappozzo et al., 1997); the positive value represented extension, external rotation, and abduction in three planes on knee movement and the negative value represented the opposite.

### Variables

Peaks of vGRF force, relevant loading rates, and impulse were used to represent performance in this study. Knee kinematics, such as angles and angular velocities in three planes and knee kinetics, such as joint moments, joint powers were used to evaluate knee movements. The movement consistency was evaluated by approaching velocity to the force plate. The jump phase was normalized to 0–100%. The normalized vGRF force (by body weight, BW), knee moment (by body mass), and knee power (by body mass) were used in the current study to exclude the variation of body mass.

### Statistics

All the discrete variables were analyzed in SPSS 24.0 (SPSS Inc., Chicago, IL, USA) and continuum curves were analyzed with SPM 1D script in Matlab 2019a (Pataky, 2010). The curves representing the holistic jump phase are normalized from biomechanics signals with 101 data points. The interesting variables were presented as mean ± SD. The mean of five trials was used to detect the differences. At the initial step, a normality test (Pipiro–Wilk's test) was performed with all parameters. If passed the test, the interesting variables were analyzed with a paired *t*-test, or the changes were determined with Wilcoxon's signed-rank test. The significant level α = *0.05* was used to determine all the differences.

## Results

**Figure 3:**
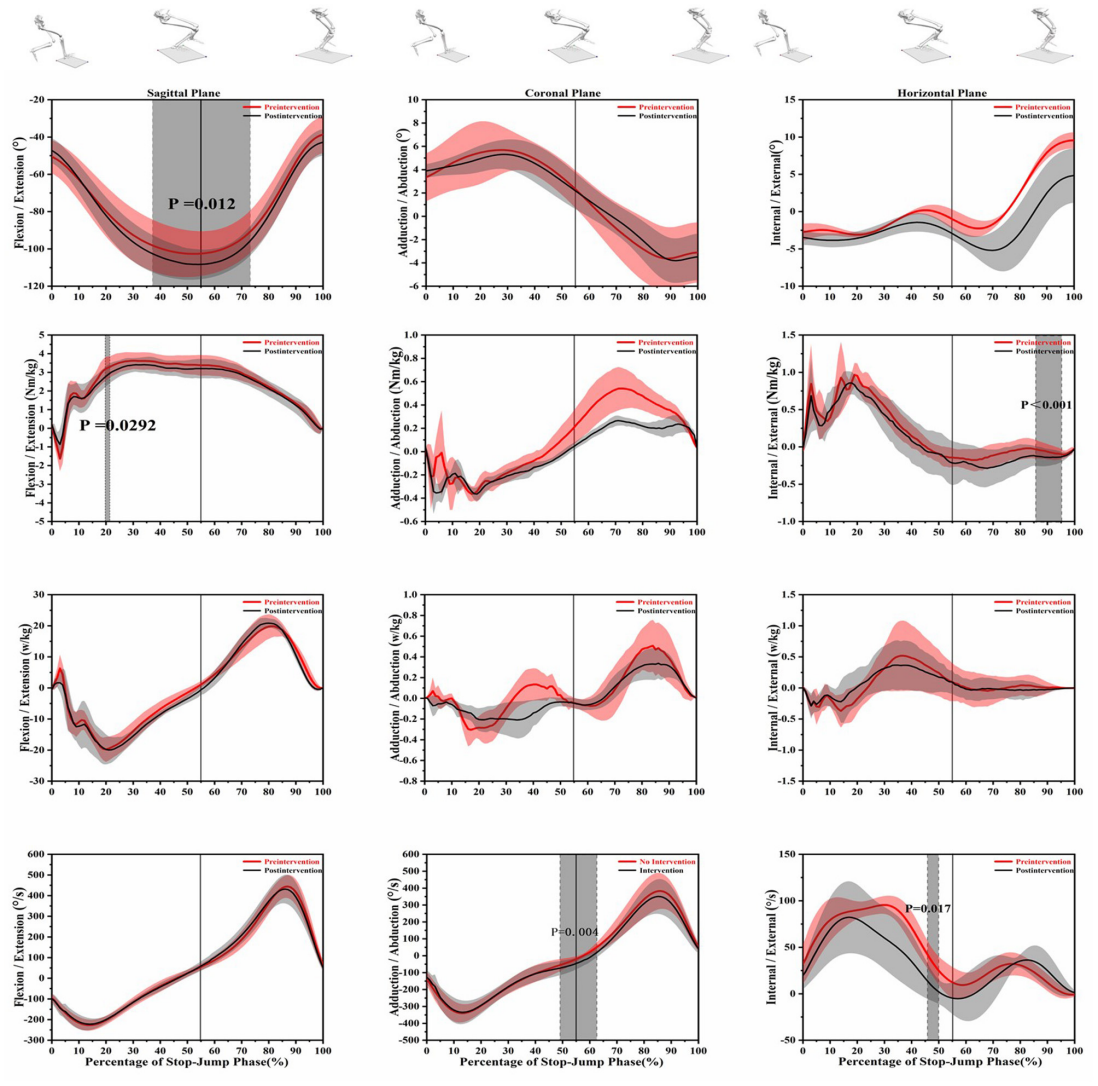
The results of statistical parametric mapping (SPM) pre and post video tasks depicting the mean angle, moment, power, angular velocity, with standard error in knee sagittal coronal, and transverse planes. The solid line at 55% represents the time of maximum knee flexation. The gray region indicates a statistical differences (*p* < 0.05).

**Figure 4:**
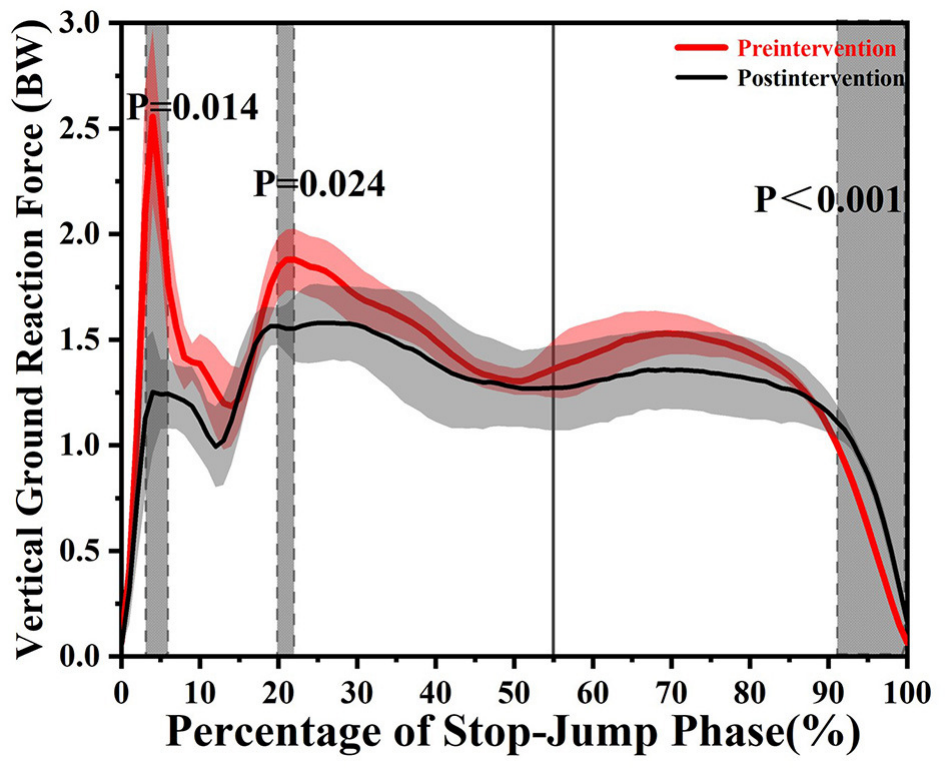
The results of statistical parametric mapping (SPM) pre and post video tasks depicting vGRF. The solid line at 55% represents the time of maximum knee flexation. The gray region indicates a statistical differences (*p* < 0.05).

Tables 1, 2 show the differences in the interesting discrete variables in the SPJ. Figures 3, 4 show the biomechanics curve differences before and after the intervention.

**Table 1:** The outcomes of vertical reaction force.

	**Before intervention**	**After intervention**	***T* value**	***p*-value**
First peak of vGRF (BW)	2.64 ± 0.32	1.42 ± 0.20	7.638	0.001^**^
Second peak of vGRF (BW)	1.91 ± 0.14	1.72 ± 0.18	2.286	0.071
Third peak of vGRF(BW)	1.54 ± 0.11	1.45 ± 0.17	1.369	0.213
Mean loading rate to the first peak (BW/s)	83.16 ± 10.61	51.58 ± 23.79	4.225	0.004^**^
Mean loading rate to the second peak (BW/s)	58.03 ± 4.37	57.31 ± 7.30	0.254	0.808
vGRF Impulse (BWs)	0.60 ± 0.07	0.53 ± 0.04	1.712	0.131

** *indicates a significance with (p < 0.01)*.

**Table 2:** The biomechanics of knee movements during spike jump (SPJ).

	**Before intervention**	**After intervention**	***T* value**	***p*-value**
Approaching velocity (m/s)	0.49 ± 0.07	0.46 ± 0.11	0.751	0.477
Initial contact flexion angle (°)	−47.23 ± 6.64	−50.53 ± 8.93	2.169	0.049^*^
Maximal flexion angle (°)	−102.90 ± 12.36	−108.53 ± 8.15	3.242	0.006^**^
Peak of flexion angular velocity (°/s)	−240.93 ± 26.62	−237.17 ± 27.67	0.848	0.413
Peak of extension angular velocity (°/s)	449.21 ± 59.88	435.85 ± 72.70	−0.073	0.943
Peak of extension moment (Nm/kg)	3.75 ± 0.48	3.51 ± 0.43	3.962	0.001^**^
Peak of flexion moment (Nm/kg)	−1.86 ± 0.63	−1.19 ± 0.55	−3.711	0.002^*^
Peak of flexion power (W/kg)	−20.46 ± 3.84	−20.52 ± 4.71	−0.677	0.518
Peak of extension power (W/kg)	20.36 ± 3.47	21.26 ± 1.60	0.249	0.811
Peak of abduction angle (°)	5.36 ± 1.29	6.08 ± 2.03	−1.238	0.304
Peak of adduction angle (°)	−3.81 ± 1.91	−3.64 ± 2.68	−0.353	0.747
Peak of abduction angular velocity (°/s)	385.99 ± 106.10	354.53 ± 103.38	0.989	0.340
Peak of adduction angular velocity (°/s)	−343.37 ± 52.92	−338.13 ± 69.47	0.307	0.763
Peak of abduction moment (Nm/kg)	0.55 ± 0.18	0.29 ± 0.05	3.069	0.037^*^
Peak of adduction moment (Nm/kg)	−0.49 ± 0.07	−0.48 ± 0.12	−0.081	0.939
Peak of abduction power (w/kg)	0.37 ± 0.13	0.60 ± 0.17	−2.258	0.087
Peak of adduction power (w/kg)	−0.29 ± 0.10	−0.40 ± 0.14	1.166	0.308
Peak of external rotation angle (°)	9.57 ± 1.33	4.84 ± 4.46	2.610	0.121
Peak of internal rotation angle (°)	−3.51 ± 1.08	−6.09 ± 1.59	1.961	0.189
Peak of external rotation angular velocity (°/s)	101.15 ± 9.16	95.83 ± 37.29	0.288	0.785
Peak of internal rotation angular velocity (°/s)	−3.41 ± 5.23	−21.41 ± 10.82	5.209	0.003^**^
Peak of external rotation moment (Nm/kg)	1.34 ± 0.31	1.08 ± 0.19	2.348	0.041^*^
Peak of internal rotation moment (Nm/kg)	−0.24 ± 0.12	−0.37 ± 0.22	2.813	0.018^*^
Peak of external rotation power (w/kg)	0.56 ± 0.55	0.88 ± 0.34	1.900	0.099
Peak of internal rotation power (w/kg)	−0.59 ± 0.14	−0.44 ± 0.22	−1.524	0.171

* *indicates statistical difference (p < 0.05).*

** *indicates a significance with (p < 0.01)*.

### Knee Kinematics Outcomes

Only increased knee flexion angle (31.17–73.19%, *t* = 2.611, and *p* = 0.012) and increased adduction angular velocity (49.07–62.64%, *t* = 3.148, and *p* = 0.004) were observed after the intervention in all interesting variables. Figure 3 shows a smaller angular velocity of external rotation after the video task (45.85–49.96%, *t* = 5.199, and *p* = 0.017). As listed in Table 2, the initial contact knee flexion angle (*t* = 2.169 and *p* = 0.049), the peak of knee flexion angle (*t* = 3.242 and *p* = 0.006), and the peak of internal angular velocity (*t* = 5.209 and *p* = 0.003) increased significantly after video task intervention, while the difference was not found about other discrete variables.

### Knee Kinetics Outcomes

As shown in Figure 3, video intervention increased the knee flexion moment (19.72–21.38%, *t* = 0.029, and *p* = 0.029) and knee external rotation moment (85.55–95.06%, *t* = 4.214, and *p* < 0.001), respectively. However, video task did not alter the abduction or adduction moment or power. As shown in Figure 4, the intervention decreased the vGRF in two stages (3.13–5.94%, *t* = 4.096, and *p* = 0.014; 19.83–21.97%, *t* = 4.096, and *p* = 0.024); however, an increased vGRF region (91.43–100%, *t* = 4.096, and *p* < 0.001) was observed. As listed in Table 2, video task decreased the knee extension/flexion moment (*t* = 3.962, −3.711 and *p* < 0.001, *p* = 0.002); the video protocol reduced the peak of knee abduction moment (*t* = 3.069 and *p* = 0.037) and increased the peak of knee internal rotation moment (*t* = 2.813 and *p* = 0.018). Table 1 shows that the first peak of vGRF (*t* = 7.638 and *p* < 0.001) and related mean loading rate (*t* = 4.225 and *p* = 0.004) are lower than before intervention.

## Discussion

The central nervous system can integrate signals from multiple sources of sensors, such as the vision to control and govern body movements (Horak and Macpherson, 2010). According to Wulf et al. (2002) enhancing internal attention enables athletes to increase their active control; athletes can consciously avoid dangerous actions during exercise, thereby reducing the risk of sports injuries (Wulf et al., 2001). The internal mechanism of this reaction can be explained as the mechanism of neurons activates to recognize relevant information when the top-down system is ready to respond to the target (Lohse and Sherwood, 2011). The objective of this research was to determine the effect of video tasks on knee movements during volleyball SPJs in male athletes. The observations in the current study partly support the hypothesis that the video task procedure would decrease the risk of knee injuries in volleyball SPJ, but it does not increase the vGRF impulse.

The finding that no difference in the approaching velocity validates that the movement is consistent for the same subject. Hence, the differences from the variations of movement can be neglected. The findings on vGRF through SPM 1D that video intervention decreases the vGRF in some stages is supported by the study (Cronin et al., 2008). Some studies explored the influence of internal attention on the peak of vGRF wherein reduced maximal force was reported after the instructions directing attention internally (Marchant et al., 2009). Wulf et al. (2002) reported that the jumping height was not affected by video intervention, which is consistent with the observation in the current study. The decrease in the first peak of vGRF along with the near region (3.13–5.94%) and its loading rate after the instructions directing attention internally reported in this study is presumably because body segments are affected more by the internal focus according to a previous study (Vance et al., 2004). It is reported that increased peak of vGRF is associated with the knee injury risk (Bates et al., 2013, Cortes et al., 2007). The first peak and related loading rate are generated in a passive stage for the subject; and, subjects struck more smoothly, thus, the risk of lower limbs would decrease according to current findings. Video intervention in the current study did not affect the other vGRF peaks (second and third) by discrete points. However, the SPM 1D method indicated that vGRF in the stage (19.83–21.97%) around the second peak decreased whereas vGRF in the stage (91.43–100%) around the last phase increased. The vGRF decreased from 19.83 to 21.97% represents that the direction of attention leans to the interior according to conscious motor control. As the stage is a braking phase, this attention strategy may lower the knee injury risk by a lower knee affordance. The increase of vGRF around the last phase implies that participants would obtain more power from the intervention in the last phase since the last phase is the propulsion phase in the SPJ. Therefore, the observations on vGRF suggest that the intervention protocol used in the current study has the potential to lower the knee injury risk and do not affect the sport performance negatively.

Of the kinematics of knee movement, the discrete point analysis shows that the video intervention can increase the contact angle, the peak of flexion angle; the SPM 1D analysis shows a more flexion knee in the phase (31.17–73.19%) (Figure 3). Meanwhile, in accordance with the findings of knee kinematics, the flexion/extension moment was found decreased, which all indicates that video intervention can result in a more flexion body posture when athletes landing on the force plate. The findings in the current study are consistent with the previous study (Yu et al., 2006). There have been numerous studies pertaining to cadavers and movement that indicate the extra valgus load of the knee is closely related to the strain of knee (Kanamori et al., 2000, 2002; Lloyd and Buchanan, 2001; Fukuda et al., 2003; Noyes et al., 2015), which is deleterious for the knee.

The discrete point analysis showed that the video intervention can decrease the peak of abduction moment and the peak of extension moment when athletes performing the SPJ. The SPM 1D analysis showed a phase (49.07–62.64 %), wherein increased angular velocity of adduction was found during the SPJ after the video intervention. A previous study reported several times increase of ACL load resulted from increased abduction moment (Markolf et al., 1995). Another study reported that verbal guidance can decrease the abduction affordance in a stop-jump (Yang et al., 2018), which is accordant with the current study. Hence, the video protocol used in the current study has the potential to reduce the load of ACL during the jump. Numerous studies pointed that extension moment is closely related to the sheer force of the knee in the anterior-posterior direction (Markolf et al., 1995; Sell et al., 2007; Wang et al., 2010). Yu et al., reported a proportional relationship between extension moment and anterior-posterior tibia shear force (Yu et al., 2006). The studies on cadavers indicate that sheer force of the knee results in acute knee injury (Markolf et al., 1995). Therefore, the decreased abduction and extension moment by discrete point analysis indicates that video intervention can lower the knee risk of injury. Furthermore, the SPM 1D analysis also indicates that the intervention can increase adduction knee velocity in the phase (49.07–62.64 %) may produce more power to facilitate the jump.

The discrete points pointed out that the intervention can enhance the internal rotation velocity of the knee and the internal rotation moment; however, the smaller peak of the external moment of the knee was observed after the intervention. The pattern of knee external rotation in landing is associated with knee injuries (Myer et al., 2004). A decreased region (45.85–49.96%) was found after intervention on knee external rotating velocity, which may contribute to lower the risk of knee, but further identification are needed in the future. A region (85.55–95.06%) wherein a larger external rotation moment of knee was found. The region is the last phase of the take-off so the larger external rotation moment suggests that this video procedure would increase the power of take-off during the last phase, which is a coincidence with the result of vGRF above. Overall, combined with the findings in all three planes, it is presumably suggested that the video task procedure would decrease the risk of the knee in SPJ without undermining the performance.

As a way to improve the focus of internal attention, video tasks have an impact not only on the biomechanics of movements but also on the action controlling process. In addition, previous research has demonstrated that internal focus of attention will lead to increased muscle activities, and increased muscle activities will evoke a series of changes in the process of movement control (Zachry et al., 2005). Hence, further exploration of muscle activities will be necessary for the future.

## Conclusion

This study indicates that knee biomechanics in the volleyball SPJ may be positively influenced by video tasks. The video task can be considered as a way to strengthen the active control of the lower limbs of athletes, which improves the original movement strategies. Furthermore, the results indicated that the sports performance may not be negatively affected by the video task protocol. Therefore, the video task presumably can abate the occurrence of knee injuries in volleyball SPJ. The video intervention can be applied in the daily training of male volleyball athletes. Further validation especially in the exercise effect is needed in the future.

## Limitations

The first limitation of this study is that the current study did not include any electromyography data. Hence, we cannot discuss the muscle activities affected by video feedback protocol, which has the chance to affect the conclusions due to the complexity of motor control. Therefore, electromyography data are needed in the future. Pertaining to this study design, a control group and a larger sample size would be helpful to validate the conclusions in future studies. The next one is that the data in the current study did not support the discussion on the exercise effect and indeed, the exercise effect is different from the immediate effect. For the application in training, the exercise effect is needed to be validated.

## Theoretical and Practical Implications

Knee injuries are common in volleyball, especially when players perform SPJs. Video feedback may be the simplest form of motor skill learning, which has the potential to contribute to injury prevention. Some studies suggested that verbal instructions given to participants can decrease the peak of vGRF in landing (Cronin et al., 2008; Milner et al., 2012), but few studies explored the take-off phase which is also vital to knee injuries and sports performance. The first contribution is to evaluate the effects of video tasks in the take-off phase in volleyball SPJ, which is a key to spike successfully. The next contribution is that we adopt the SPM method in this study, which can evaluate the differences between curves and whereby we can detail the effects further than before. The last contribution is that the developed video task in the current study is simple and time-saving, and hence can be carried out in training.

## Data Availability Statement

The raw data supporting the conclusions of this article will be made available by the authors, without undue reservation.

## Ethics Statement

The studies involving human participants were reviewed and approved by Insititute of sports science institutional review board, Ningbo University. The patients/participants provided their written informed consent to participate in this study.

## Author Contributions

XL, HH, and AW: conceptualization. XL and AW: methodology. JL, HS, and AW: validation. HS and AW: review and editing. All authors were involved in the final direction of the study and agreed to the published version of the manuscript.

## Conflict of Interest

The authors declare that the research was conducted in the absence of any commercial or financial relationships that could be construed as a potential conflict of interest.

## Publisher's Note

All claims expressed in this article are solely those of the authors and do not necessarily represent those of their affiliated organizations, or those of the publisher, the editors and the reviewers. Any product that may be evaluated in this article, or claim that may be made by its manufacturer, is not guaranteed or endorsed by the publisher.
